# miR-34a inhibits pancreatic cancer progression through Snail1-mediated epithelial–mesenchymal transition and the Notch signaling pathway

**DOI:** 10.1038/srep38232

**Published:** 2017-02-01

**Authors:** Yan Tang, Yong Tang, Ying-sheng Cheng

**Affiliations:** 1 Department of Radiology, Shanghai Jiao Tong University Affiliated Sixth People’s Hospital, Shanghai, 200233, China; 2 Department of Cardiology, Xinhua Hospital, Shanghai Jiaotong University School of Medicine, Shanghai, China

## Abstract

Epithelial–mesenchymal transition (EMT) and Notch signaling are important for the growth and invasion of pancreatic cancer, which is a leading cause of cancer-related deaths worldwide. miR-34a has been shown to play pivotal roles in the progression of several types of cancer. However, little is known about the regulatory mechanisms of miR-34a in pancreatic cancer processes. The aim of this study was to determine whether miR-34a has negative effects on pancreatic cancer and whether these effects are related to EMT and Notch signaling. *In vitro*, we demonstrated that miR-34a inhibited, while miR-34a inhibitors enhanced, migration and invasion of pancreatic cancer cell lines (PANC-1 and SW-1990).These effects were reversed by Snail1 overexpression or Snail1 shRNA. Furthermore, the anti-apoptotic effects of the miR-34a inhibitors in pancreatic cancer cells were abrogated by Notch1 shRNA. Luciferase reporter assays revealed that the Snail1 and Notch1 genes were direct targets of miR-34a. *In vivo*, we also demonstrated that miR-34a inhibited pancreatic cancer growth by decreasing Snail1 and Notch1 expression. Therefore, our results indicate that miR-34a inhibits pancreatic cancer progression by post-transcriptionally regulating Snail1 and Notch1 expression.

Pancreatic cancer, which is a leading cause of cancer-related deaths worldwide, is characterized by poor prognosis and rapid progression. Although diagnostic and therapeutic measures have improved, survival remains dismal, with an overall five-year survival rate of 8% in America^1^. Therefore, identification of the biological mechanisms underlying the development of pancreatic cancer is urgent.

Previous studies have indicated that epithelial–mesenchymal transition (EMT) and Notch signaling are important for the growth and invasion of pancreatic cancer^2^,^3^,^4^. EMT is a reversible program that is defined as the transition from epithelial cells to mesenchymal cells^5^. EMT plays a key role in embryonic development, tissue regeneration and cancer metastasis. The loss of E-cadherin protein and the acquisition of mesenchymal markers, such as N-cadherin, vimentin and fibronectin, are characteristic of the EMT program^3^. EMT is induced by several signaling pathways, including transforming growth factor-β (TGF**-**β) and Notch. Emerging evidence has indicated that Notch signaling and EMT have many functional overlaps by regulating cell survival^6^,^7^. In TGF**-**β-mediated EMT, the transcription factors Snail or ZEB repress the expression of E-cadherin and induce N-cadherin^8^,^9^. Therefore, the regulation of EMT and Notch signaling is closely related to pancreatic cancer treatment.

Studies have shown that miR-34a plays pivotal roles in cancer suppression. miR-34a negatively regulates multiple target genes involved in cancer cell growth, proliferation, apoptosis and invasion. Researchers noted that miR-34a inhibits breast cancer cell growth by repressing the Wnt1-MTA1-β-catenin axis^10^. The crosstalk between miR-34a and p53 has an important tumor suppressor effect^11^. A recent report found that the overexpression of miR-34a induced pancreatic cancer cell apoptosis and inhibited cell growth^12^. Furthermore, serum miR-34a is potential biomarker of pancreatic ductal adenocarcinoma^13^. However, the exact regulatory mechanisms of miR-34a in pancreatic cancer processes remain unclear. Therefore, we aimed to investigate whether the negative effects of miR-34a on pancreatic cancer are related to EMT and Notch signaling.

## Results

### miR-34a inhibits pancreatic cancer cell migration and invision and induces apoptosis

**Figure 1:**
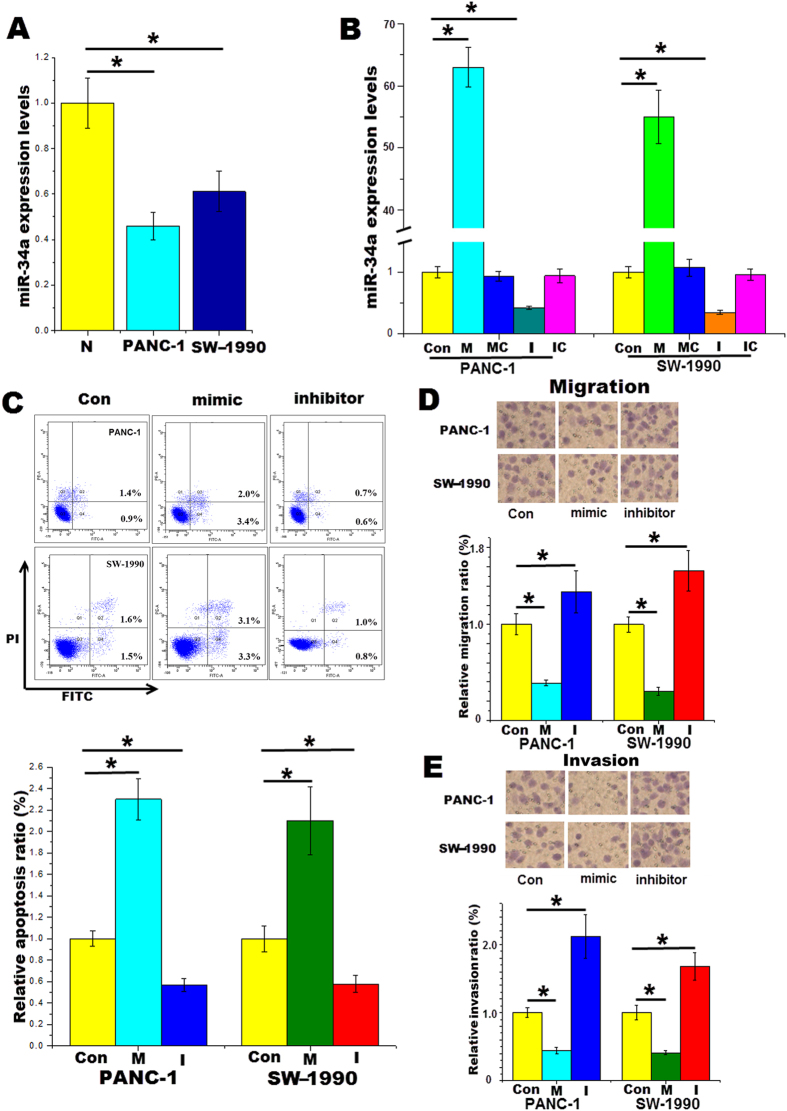
miR-34a inhibits pancreatic cancer cell migration and invasion and induces apoptosis. (**A**) RT-PCR results demonstrate that the expression levels of miR-34a were significantly lower in pancreatic cancer cell lines PANC-1 and SW-1990 than normal HPDEC. (**B**) Changes in miR-34a levels in the cell lines 24 h after transfection with the vehicle, the miR-34a mimics, the mimic control, the miR-34a inhibitors or the inhibitor control. (**C**) Following 24 h transfection of pancreatic cancer cell lines with miR-34a mimics or inhibitors, cells were stained with FITC Annexin V and PI for FC analysis. (**D**) Cell migration assays were performed. (**E**) Cells invasion assays were performed. *Con* control, *M* the miR-34a mimics, *I* the miR-34a inhibitors.*p < 0.05.

The expression levels of miR-34a were significantly lower in the pancreatic cancer cell lines PANC-1 and SW-1990 than those of normal pancreatic epithelial cells as determined by qRT-PCR (Fig. 1A), indicating that miR-34a may be restricted in pancreatic cancer. This phenomenon was consistent with previous reports^14^,^15^, which also found that the expression of miR-34 was decreased in pancreatic tumors. Subsequently, the miR-34a mimics or inhibitors were transfected into pancreatic cancer cell lines. The miR-34a mimics (100 nM) upregulated, whereas the miR-34a inhibitors (100 nM) downregulated, the miR-34a expression in both PANC-1 and SW-1990 cells (Fig. 1B).

To determine the effects of miR-34a in pancreatic cancer, we transfected the miR-34a mimics or inhibitors into pancreatic cancer cell lines. Subsequently, cell migration, invasion and apoptosis assays were performed after 24 h. We observed that the miR-34a mimics significantly enhanced the apoptosis ratio of PANC-1 as determined by flow cytometry (3.4% vs 0.9%), but the inhibitors decreased this ratio (0.6% vs 0.9%). A similar trend was also observed in SW-1990 cells 24 h after transfection with the miR-34a mimics or inhibitors (Fig. 1C). Furthermore, in both PANC-1 and SW-1990 cells, the number of the migrated and invaded cells following miR-34a overexpression was decreased. Inhibition of miR-34a expression increased these cell numbers, as expected (Fig. 1D,E).

### Snail1 is a target gene of miR-34a

**Figure 2:**
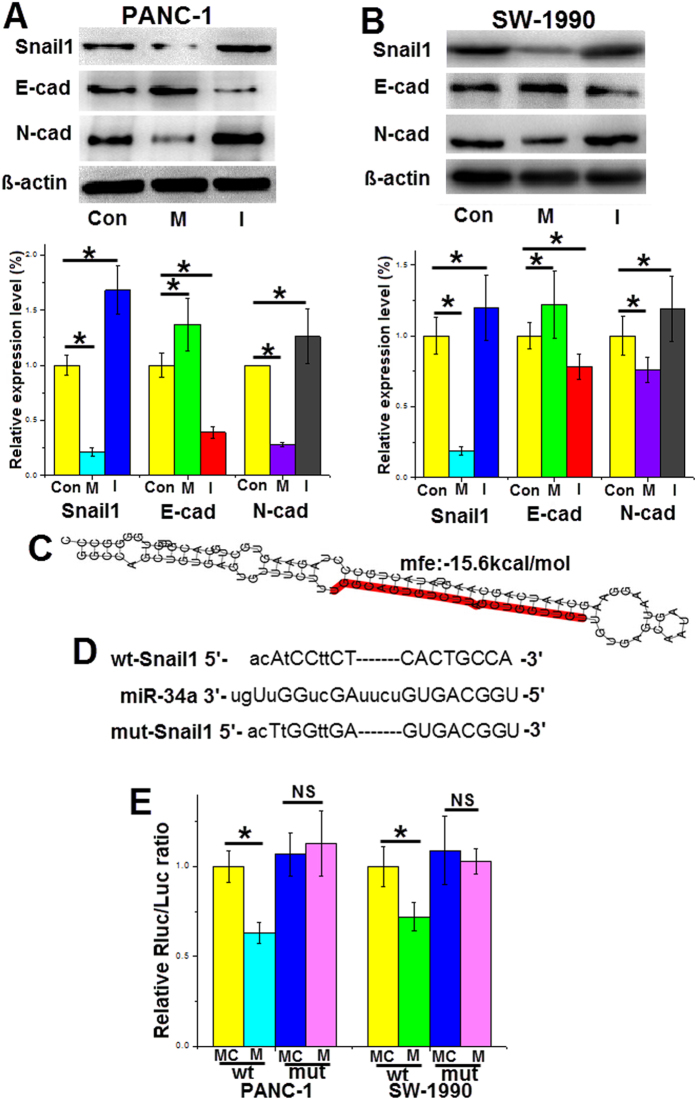
Snail1 is a target gene of miR-34a. (**A,B**) Forty-eight hours after transfection with miR-34a mimics or inhibitors in pancreatic cancer cell lines, the protein levels of Snail1, E-cadherin and N-cadherin in the cells were measured. (**C**) The miR-34a binding sites in the 3′-UTR of the Snail1 gene. (**D**) The wild type and mutant binding sites for miR-34a in the 3′-UTR of the Snail1 gene. (**E**) The relative luciferase activity. *Con* control, *M* the miR-34a mimics, *I* the miR-34a inhibitors, *MC* mimic control, *IC* inhibitor control, *wt* wild type, *mut* mutant type. *p < 0.05. The full length blots are presented in Supplementary Figs S1 and S2.

Decreases in the adhesion molecules of endothelial cells are associated with pancreatic cancer invasion. Several transcription factors involved in EMT, such as Snail1, repress the genes encoding adhesion molecules, promoting cancer cell invasion^16^. Here, we found that the protein expression levels of Snail1 in pancreatic cancer cell lines were significantly repressed after transfecting the cells with the miR-34a mimics for 48 h. Furthermore, we demonstrated expression changes in proteins characteristic of the EMT program. In both PANC-1 and SW-1990 cells, increased expression of miR-34a upregulated the protein levels of E-cadherin and downregulated the protein levels of N-cadherin (Fig. 2A,B).

Using several publicly available target prediction web sites (miRanda, TargetScan and miRBase), we identified Snail1 as a potential direct target of miR-34a. The 3′-UTR of the Snail1 gene has binding sites for miR-34a, and the MFE value of hybridization determined by RNAhybrid software was −15.6 kcal/mol (Fig. 2C). To further confirm this prediction, the miR-34a mimics and the luciferase vectors containing the wild-type or mutant 3′-UTR binding sites of the Snail1 gene were co-transfected into PANC-1 and SW-1990 cells. The luciferase activity of wild-type plasmid was significantly decreased, but the mutant plasmid was not suppressed (Fig. 2D,E). These data suggests that Snail1 is a direct target gene of miR-34a.

### miR-34a regulates Snail1-mediated EMT in pancreatic cancer

**Figure 3:**
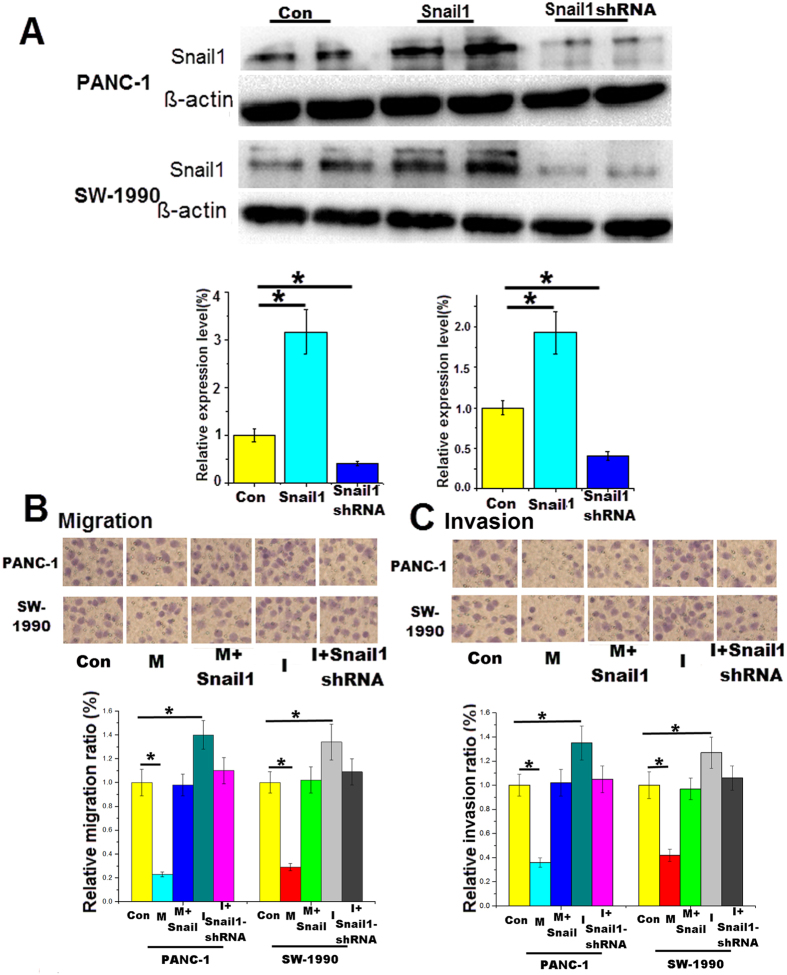
miR-34a regulates the Snail1-mediated EMT program in pancreatic cancer. (**A**) Changes in Snail1 protein levels in the cell lines 48 h after transfection with the Snail1 overexpression plasmid or the Snail1-shRNA. Cells were co-transfected with the miR-34a mimics and the Snail1 overexpression plasmid or co-transfected with the miR-34a inhibitors and the Snail1-shRNA for 24 h, and (**B**) the migration assay was performed; (**C**) the invasion assay was performed. *Con* control, *M* the miR-34a mimics, *I* the miR-34a inhibitors. *p < 0.05. The full length blots are presented in Supplementary Fig. S3.

Previous studies have demonstrated that the EMT program plays a key role in tumor invasion and that Snail1 is an important transcription factor in this process^17^,^18^. First, the pancreatic cancer cells were transfected with the Snail1 overexpression plasmid or Snail1-shRNA for 48 h. The western blotting results revealed the protein expression changes with Snail1 gene overexpression or silencing (Fig. 3A). Next, cell migration and invasion assays were performed using 8-μm pore size Transwell plates. Migration and invasion were decreased in cancer cells overexpressing miR-34a, and the effects were restored by Snail1 overexpression. The promotion of cell migration and invasion by the miR-34a inhibitors was reversed by the Snail1 shRNA (Fig. 3B,C). Taken together, the results showed that miR-34a inhibits pancreatic cancer cell invasion by regulating Snail1, a key transcription factor of the EMT program.

### Notch1 is a target gene of miR-34a

**Figure 4:**
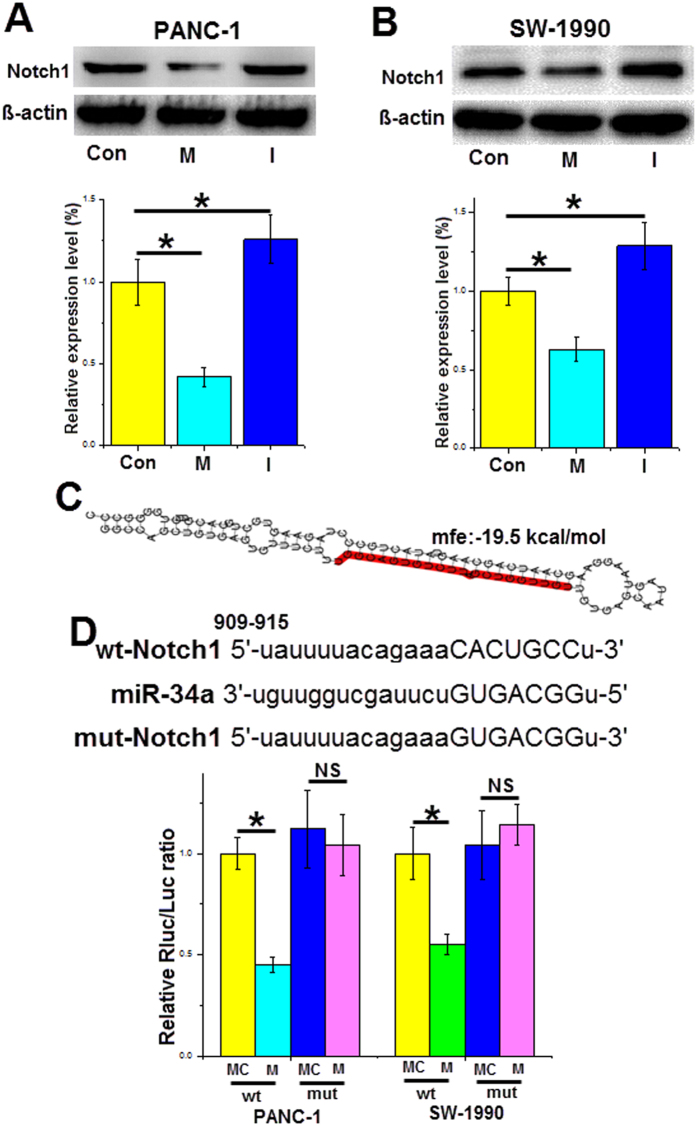
Notch1 is a target gene of miR-34a. (**A,B**) Following 48 h of transfection with miR-34a mimics or inhibitors into pancreatic cancer cell lines, the cleaved Notch1 protein levels were detected by western blotting. (**C**) The miR-34a binding sites in the 3′-UTR of the Notch1 gene. (**D**) The wild type and mutant binding sites for miR-34a in the 3′-UTR of the Notch1 gene and the relative luciferase activity. *Con* control, *M* the miR-34a mimics, *I* the miR-34a inhibitors, *MC* mimic control, *wt* wild type, *mut* mutant type. *p < 0.05. The full length blots are presented in Supplementary Figs S4 and S5.

Emerging evidence has indicated that Notch signaling plays a key role in pancreatic cancer cell proliferation and apoptosis^19^,^20^. After transfection for 48 h, Notch1 protein expression was downregulated in PANC-1 cells transfected with the miR-34a mimics (by approximately 55%) and increased in cells transfected with the inhibitors (by approximately 20%) compared to that of the control group (Fig. 4A). Similar results were found in SW-1990 cells, and the miR-34a mimics decreased the Notch1 protein expression by approximately 40%, and the miR-34a inhibitors increased the Notch1 protein expression by approximately 20% (Fig. 4B). Furthermore, we found that Notch1 may also be a direct gene target of miR-34a using target prediction sites. The 3′-UTR of Notch1 contains the binding sites for miR-34a with a −19.5 kcal/mol MEF value (Fig. 4C). A luciferase assay revealed that the miR-34a mimics decreased the luciferase activity of the wild-type Notch1 reporter plasmids; however, no suppression was found for the mutant reporter plasmids (Fig. 4D). Thus, miR-34a could directly regulate Notch1 gene expression at the post-transcriptional level.

### miR-34a inhibits pancreatic cancer cell proliferation and induces apoptosis by targeting Notch1 expression

**Figure 5:**
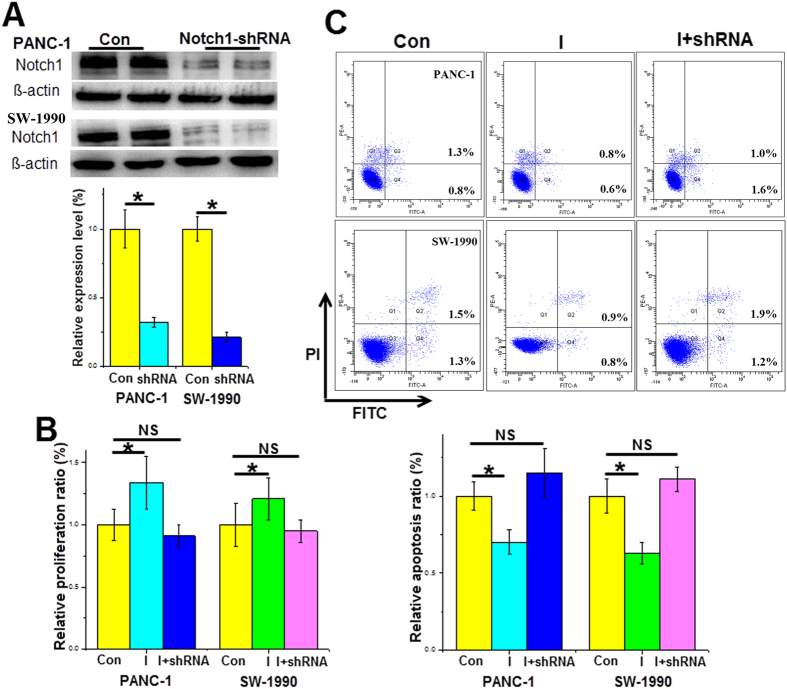
miR-34a inhibits pancreatic cancer cell proliferation and induces apoptosis by targeting Notch1 expression. (**A**) Changes in the cleaved Notch1 protein levels of the cell lines 48 h after transfection with the Notch1-shRNA. Cells were co-transfected with the miR-34a inhibitors and the Notch1-shRNA for 24 h, (**B**) proliferation was measured by the CCK-8 method, and (**C**) apoptosis was detected by the FC method. *Con* control, *I* the miR-34a inhibitors. *p < 0.05. The full length blots are presented in Supplementary Fig. S6.

As mentioned above, miR-34a could directly regulate the Notch1 gene, which is involved in cell proliferation and apoptosis. After transfection with Notch1-shRNA for 48 h, the Notch1 protein levels were significantly downregulated in both the PANC-1 and SW-1990 cells (Fig. 5A). Next, CCK-8 assays demonstrated that the pro-proliferation effect of the miR-34a inhibitors was partly reversed after Notch1 gene silencing (Fig. 5B). The flow cytometric analysis, as shown in Fig. 1, indicated that the miR-34a inhibitors significantly decreased pancreatic cancer cell apoptosis by approximately 20%.However, downregulated expression of Notch1 abrogated the anti-apoptotic effect of the miR-34a inhibitors on pancreatic cancer cells (Fig. 5C). Based on the above results, we demonstrated that Notch1 is crucial to miR-34a’s function as a tumor suppressor.

### Notch1 affect miR-34a expression

**Figure 6:**
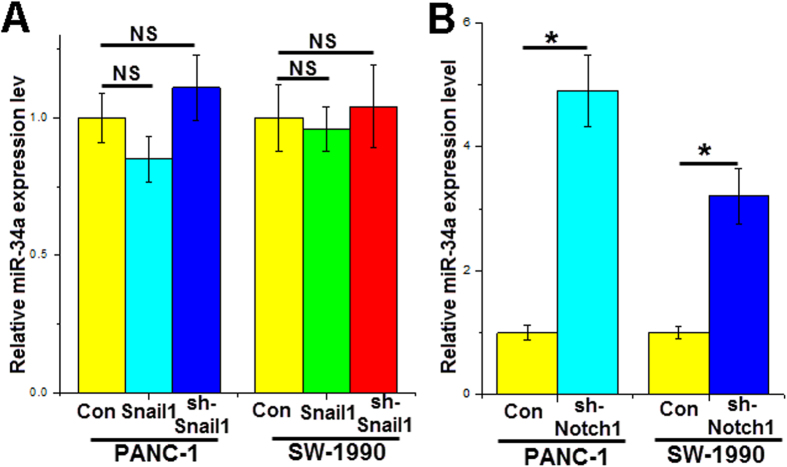
Snail1 and Notch1 affect miR-34a expression. The miR-34a expression levels were detected by RT-PCR after transfection with the Snail1 overexpression plasmid or Snail1-shRNA (**A**), and Notch1-shRNA (**B**) in cell lines for 24 h. *p < 0.05.

The Notch1 and Snail1 genes are important for cancer cell growth and invasion. miR-34a could inhibit pancreatic cancer cell proliferation, induce apoptosis and decrease invasion. Therefore, we investigated whether aberrant expression of Notch1 or Snail1 could affect miR-34a expression levels in pancreatic cancer cells. We altered the Snail1 or Notch1 gene expression levels in pancreatic cancer cell lines by transfection with gene expression vectors or shRNAs for 24 h. Compared with the control group, downregulated Notch1 significantly increased miR-34a expression (Fig. 6B) in PANC-1 cells, while upregulated or downregulated Snail1 did not change the miR-34a level (Fig. 6A). Similar results were observed in SW-1990 cells, in which Notch1 silencing increased miR-34a expression by approximately 200% (Fig. 6B). These data suggest that there is a positive feedback loop between miR-34a and the Notch1 gene in the progression of pancreatic cancer.

### miR-34a functions *in vivo*

**Figure 7:**
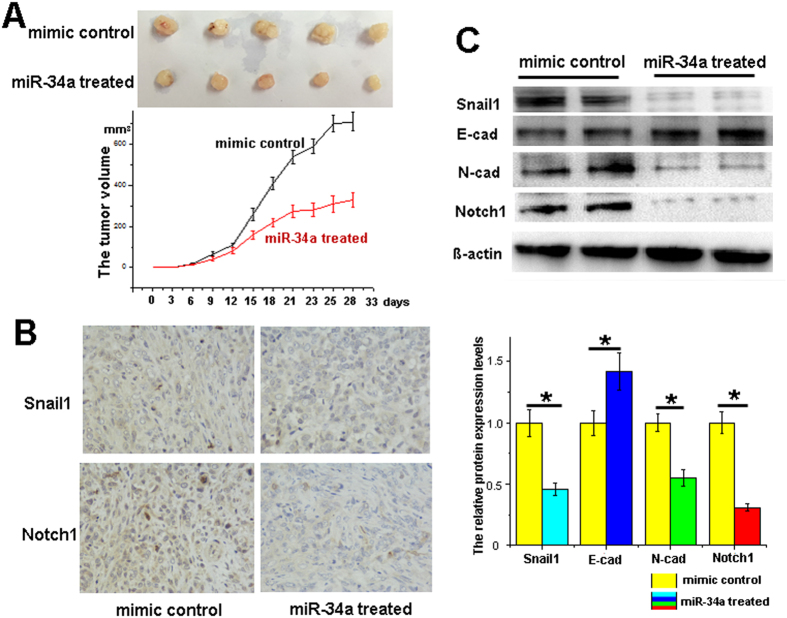
miR-34a functions *in vivo*. After the miR-34a mimic- or mimic control-transfected PANC-1 cells were implanted into the nude mice for 5 weeks, (**A**) tumors were observed, and the tumors volumes were measured every 3 days. The protein expression of Snail1 and Notch1 was detected by IHC (**B**) and western blotting (**C**). *p < 0.05. The full length blots are presented in Supplementary Fig. S8.

To examine the regulatory effects of miR-34a on pancreatic cancer *in vivo*, the PANC-1 cells were pretreated with miR-34a mimics or mimic control for 24 h *in vitro*. Then the miR-34a mimic- or mimic control-transfected PANC-1 cells were administered to 4-week-old BALB/c nude mice. The formulated miR-34a mimics or mimic control were administrated by a dose of 100 nM twice a week via tail-vein injection and the tumor volumes were measured every 3 days. After 5 weeks, the mice were sacrificed, and the tumor tissues were collected. In the miR-34a mimic-treated group, the tumor volumes were significantly smaller than those of the control group (Fig. 7A). The levels of miR-34a in the miR-34a treated group were significantly higher than the control group (Supplementary Fig. S7). Furthermore, the results of IHC and western blotting demonstrated that the expression of Snail1 and Notch1 decreased with miR-34a overexpression (Fig. 7B,C).

## Discussion

miR-34a is a member of the conserved miR-34 family, which includes miR-34a, miR-34b and miR-34c^21^,^22^. The miR-34a coding sequence differs from that of the other miR-34 family members. miR-34a is encoded by one transcript, while miR-34b and miR-34c are encoded by another transcript. All the members of the miR-34 family have been reported to be tumor suppressor miRNAs. The expression level of miR-34a in normal tissues (except lung tissues) is much higher than that of miR-34b and miR-34c^21^. The functions of the three types of miR-34 are diverse. The regulatory effects of miRNAs on tumor growth, apoptosis, migration and invasion are closely related to the abundance of the specific miRNA^23^. Therefore, many studies have focused on the function of miR-34a in various tumor tissues^10^,^12^,^22^. However, previous reports did not reveal the crosstalk between miR-34a, Notch signaling and the EMT program. In our study, we found that decreased miR-34a levels in pancreatic cancer cell lines are associated with stronger cancer activity, such as rapid growth and increased migration and invasion.

Notch signaling is important for cell proliferation and apoptosis and is activated by binding to the specific ligands (Jag1 and 2 or DLL1, 3, and 4)^24^,^25^. Four types of Notch genes, including Notch1, 2, 3, 4, have been identified thus fa r^2^. Recently, emerging evidence has shown that Notch signaling and the EMT program have some functional overlaps. The transcription factors of EMT, such as ZEB1, ZEB2, Snail1 and Snail2, control EMT phenotype, which triggers cancer cell invasion. Several researchers have reported that Notch signaling activators could also induce the EMT program in different cancer types^4^,^26^. Others demonstrated that the inducing transcription factors of EMT can enhance Notch activation^27^,^28^. Here, we showed that miR-34a regulates both Notch1 and the EMT activator Snail1 at the post-transcriptional level, which affects pancreatic cancer cell processes.

Previous studies have suggested that miR-34a exerts its function by targeting the Notch1 gene in different types of cells. Roy S *et al*. reported that the overexpression of miR-34a led to a marked reduction in Notch1 expression in colon cancer^29^. Chen Q *et al*. showed that miR-34a inhibited vascular smooth muscle cell proliferation and migration by modulating Notch1 gene expression^30^. Xia J *et al*. demonstrated that a natural compound, genistein, increased miR-34a expression levels, leading to decreased Notch1, which inhibited cell growth and induced apoptosis^31^. However, it remains unknown whether the Notch1 gene is a target of miR-34a or whether overexpression of Notch1 regulates miR-34a in pancreatic cancer. In our study, we showed that miR-34a and Notch1 repress the expression of each other and constitute a positive feedback loop in pancreatic cancer cells. Overexpression of Notch1 downregulated miR-34a expression levels in pancreatic cancer, and the downregulation of miR-34a alleviated its inhibition of the Notch1 gene, resulting in increased Notch1 protein expression. This indicates that Notch1 is not only a target gene of miR-34a but also participates in the regulation of miR-34a expression. Our findings contribute to the elucidation of the mechanism underlying Notch1 functions in the progression of pancreatic cancer.

Snail1 is a zinc-finger protein that activates the EMT program by inducing transcription of mesenchymal genes (e.g., N-cadherin) and suppressing transcription of epithelial genes (e.g., E-cadherin, occludin)^32^,^33^. The functions of Snail1 have been widely investigated in previous studies. The human Snail1 gene is composed of 264 amino acids and is located on chromosome 20q13.2^34^. High expression levels of Snail1 in cancer patients are usually associated with poor prognosis. The transcriptional level of Snail1 could be regulated by several factors that interact directly with the Snail1 promoter, such as hypoxia-induciblefactor-1α (HIF-1α), STAT3, and NF-κB^35^,^36^. Although these factors bind to different sites in the Snail1 promoter, they all enhance Snail1 gene expression. Snail1 protein is quickly degraded in the cytoplasm, and the nuclear localization signals direct Snail1 into the nucleus. E-cadherin, PTEN and occlude in are all targets of Snail1. For example, Snail1 directly represses the expression of the epithelial marker E-cadherin^37^. In our study, after transfection with the miR-34a mimics for 48 h, the western blotting results revealed that downregulation of Snail1 protein was accompanied by upregulation of E-cadherin (Fig. 2). This indicates that the downregulation of Snail1 by miR-34a attenuates its inhibitory effect on expression of E-cadherin. E-cadherin is a transmembrane glycoprotein and has anti-migratory and anti-invasive properties in cancer cells. In our research, the anti-invasive effect of miR-34a was abrogated by overexpression of Snail1 (Fig. 3), which directly represses the expression of E-cadherin. Therefore, our results demonstrated that miR-34a inhibits pancreatic cancer cell invasion by post-transcriptionally regulating Snail1.

Because the EMT program activator Snail1 and the proliferation regulator Notch1 are both targets of miR-34a, miR-34a is vital to the progression of pancreatic cancer. In conclusion, our findings demonstrated that miR-34a plays a key role in inhibition of pancreatic cancer cell growth and cell migration and invasion. These anti-tumor effects could be abrogated by the overexpression of the miR-34a target genes Snail1 and Notch1.

Recently, the clinical diagnostic and therapeutic worth of the miR-34a has been discussed. Dufour A *et al*. reported that the downregulation of miR-34a in chronic lymphocytic leukemia patients has been associated with a poor prognosis^38^. Alemar B *et al*. demonstrated that the circulating miR-34a level can be used as a prognostic biomarker of pancreatic ductal adenocarcinoma^13^. These data suggested that elevated miR-34a level might be a novel therapeutic approach for cancer patients. However, lots of questions need to be answered from basic research to clinical application. The biological effects of microRNAs are microenvironment or cell-type dependent. For example, the miR-23a inhibits endothelial cell apoptosis, but promotes apoptosis of human embryonic kidney cells^39^,^40^. Furthermore, the miRNA function method is not a single-strand contact but rather a largely unknown network mode. Hundreds of target genes could be regulated by one miRNA, and one target gene could also be regulated by hundreds of miRNA^41^. Although more researches need to be done, our results may help to further elucidate the underlying mechanism of miR-34a in pancreatic cancer.

## Materials and Methods

### Cell culture

PANC-1, SW-1990 cell lines and the normal pancreatic duct epithelial cells (HPDEC) were obtained from the Cell Bank of the Chinese Academy of Science (Shanghai, China). The cells were cultured in DMEM high-glucose medium (HyClone) that contains 10% fetal bovine serum (HyClone) at 37 °C in a humidified incubator with 5% CO_2._ The cells were passaged less than twenty times.

### Quantitative real-time reverse transcription PCR

Total RNA was isolated using TRIzol reagent (Invitrogen) according to the manufacturer’s protocol. The reverse transcription reactions for the miRNA were performed using amiRcute miRNA cDNA kit (Tiangen).The SYBR kit (TaKaRa) was used for detection of miR-34a on an ABI7500 system(Applied Biosystems). U6 was used for normalization of miR-34a. The differences between groups were calculated using the 2^−ΔΔCt^ method. The sequence of the primer for miR-34a was as follows: 5′-TGGCAGTGTCTTAGCTGGTTGT-3′.The sequence of the primer for U6 was as follows: 5′-CGCTTCGGCAGCACATATACTAAAATTGGAAC-3′.

### Transfection *in vitro*

miR-34a mimics or inhibitors (RiboBio) were used to upregulate or downregulate the miR-34a expression, respectively. The Snail1 overexpression plasmid, Snail1-shRNA and Notch1-shRNA were obtained from RiboBio. All shRNAs and miR-34a mimics (100 nM) or inhibitors (100 nM) as well as the controls were transfected into the cell lines with Lipofectamine 2000 (Invitrogen). Changes in miR-34a expression were measured by qRT-PCR 24 h after transfection, and changes in Snail1 or Notch1 protein expression were determined by western blotting 48 h after transfection.

### Western blotting

Transfected cells were lysed for 30 min on ice with a cell lysissolution containing 1% PMSF. The extracted protein was separated using SDS–PAGE (Millipore), and equal amounts of protein (40 μg) were run under the same experimental conditions. Primary antibodies targeting Snail1 (1:1000 dilution; Cell Signaling), E-cadherin (1:1000 dilution; Cell Signaling), N-cadherin (1:1000 dilution; Cell Signaling) and Notch1 (1:1000 dilution; Cell Signaling) were used. β-actin (1:1000 dilution; Beyotime) served as the endogenous control. The original whole gel blots were included in the supplementary data.

### Cell counting kit-8 assay

Each hole of the 96-well plates with pretreated cells were added by10 μl cell counting kit-8 solution (CCK-8). Then incubate the plate at 37 °C in a humidified incubator with 5% CO_2._ After 1 h, we measure the absorbance at 450 nm using the microplate reader (BioTek).

### Flow cytometric analysis

A FITC Annexin V Apoptosis Detection Kit I (BD) was used to detect the apoptosis of pancreatic cancer cells. Briefly, the cells were transfected with miR-34a mimics, inhibitors or shRNAs in 6-well plates for 24 h. Subsequently, the cells were digested using 0.25% trypsin without EDTA. After cold PBS and 1X binding buffer were added, the cells were incubated in FITC-Annexin V solution and PI following the manufacturer’s instructions. The treated cells were immediately analyzed using a Beckman CoulterFC500 (Beckman).

### Histological analysis

Immunohistochemistry (IHC) analysis was performed according to standard protocols. The primary antibodies used for IHC analysis were a Snail1 antibody (1:100 dilution; Cell Signaling) and a Notch1 antibody (1:100 dilution; Cell Signaling).

### Luciferase activity assay

For dual luciferase assays, the 3′-untranslated region (UTR) of Snail1 and Notch1 containing the predicted potential miR-34a binding sites or mutated binding sites was amplified by RT-PCR and subcloned into the pmiR-REPORT^TM^ vector (RiboBio).

The miR-34a mimics and the vectors were co-transfected into PANC-1 cells for 24 h. Subsequently, the luciferase activity was measured using the Dual-Luciferase Reporter Assay System (Promega).

### Invasion and migration

Cell invasion and migration assays were performed using a 12-well Transwell plate precoated with or without Matrigel (BD). Then, 2 × 10^5^ cells were plated in the upper chamber with serum-free culture medium. The lower chamber had high-glucose DMEM medium containing 10% FBS. After incubation for 24 h, the membrane was washed with PBS, fixed with 4% paraformaldehyde and stained with 0.1% crystal violet. The number of cells was counted in five random fields of view using microscopy (Leica).

### Animal studies

The animal experiments were performed in the animal research center of Shanghai No.6 People’s Hospital and was approved by the ethics committee of Shanghai No.6 People’s Hospital (approval ID: 20160203). All the experiments were conducted in accordance with approved guidelines, including any relevant details. PANC-1 cells (1 × 10^7^) were transfected with miR-34a mimics (100 nM) or mimic controls for 24 h. Subsequently, the two groups of cells were collected and injected into 4-week-old BALB/c nude mice (n = 5 for one group). The formulated miR-34a mimics or mimic control were administrated via tail vein by a dose of 100 nM twice a week, as well as the tumor volumes were determined. After 5 weeks, the tumor tissues were detached from the sacrificed mice and were used for IHC and western blot analysis.

### Statistics

Statistic alanalysis was performed using SPSS 18 software (SPSS Inc., USA). All independent experiments were performed in triplicate. The data are presented as the mean ± standard error. The differences between two groups were tested using a paired t-test. A P-value < 0.05 was considered statistically significant.

## Additional Information

**How to cite this article**: Tang, Y. *et al*. miR-34a inhibits pancreatic cancer progression through Snail1-mediated epithelial–mesenchymal transition and the Notch signaling pathway. *Sci. Rep.* **7**, 38232; doi: 10.1038/srep38232 (2017).

**Publisher's note:** Springer Nature remains neutral with regard to jurisdictional claims in published maps and institutional affiliations.

## Supplementary Material

Supplementary Information
