# Supplementary material for: miR-34a inhibits pancreatic cancer progression through Snail1-mediated epithelial–mesenchymal transition and the Notch signaling pathway
Source: Sci Rep. 2017 Feb 1;7:38232. doi: 10.1038/srep38232 (PMC5286431; doi:10.1038/srep38232)
Supplement: Supplementary Information [file srep38232-s1.pdf]

Supplementary Information file

Title:miR-34a inhibits pancreatic cancer progression through Snail1-mediated epithelial–mesenchymal transition and the Notch signaling pathway

Authors: Yan Tang, Yong Tang, Ying-sheng Cheng.

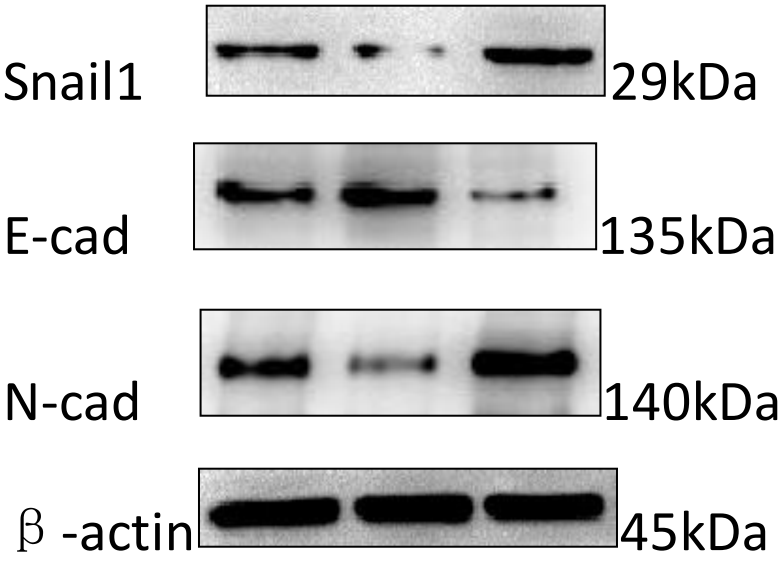

Supplementary Figure S1. Full length blots of data shown in Fig.2A

The gels were initially cut ranged from 29kd to 140 kd.

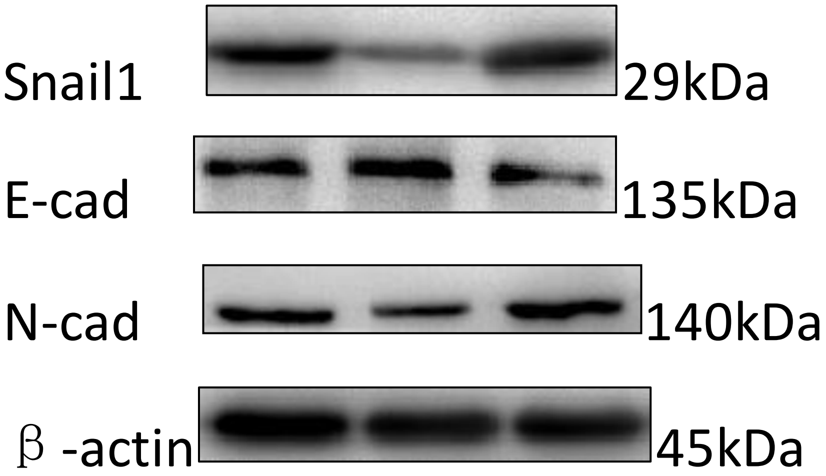

Supplementary Figure S2. Full length blots of data shown in Fig.2B

The gels were initially cut ranged from 29kd to 140 kd.

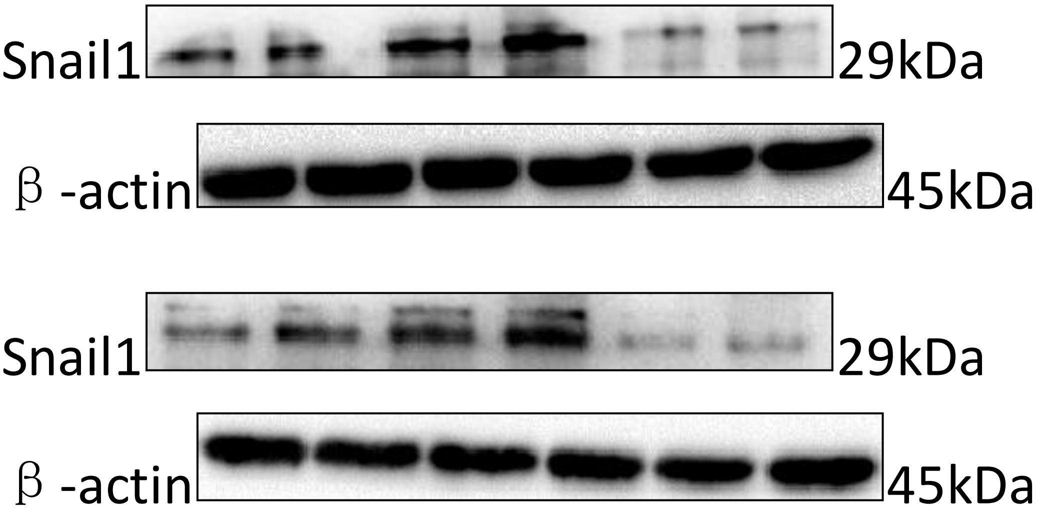

Supplementary Figure S3. Full length blots of data shown in Fig.3A

The gels were initially cut ranged from 29kd to 45 kd.

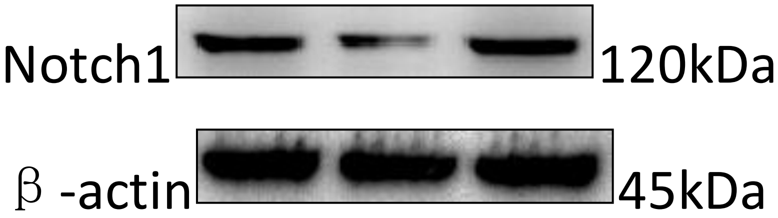

Supplementary Figure S4. Full length blots of data shown in Fig.4A

The gels were initially cut ranged from 45kd to 120kd.

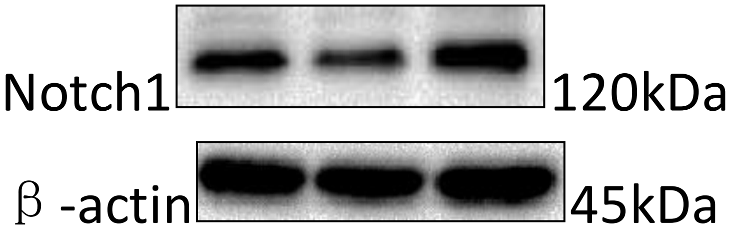

Supplementary Figure S5. Full length blots of data shown in Fig.4B

The gels were initially cut ranged from 45kd to 120 kd.

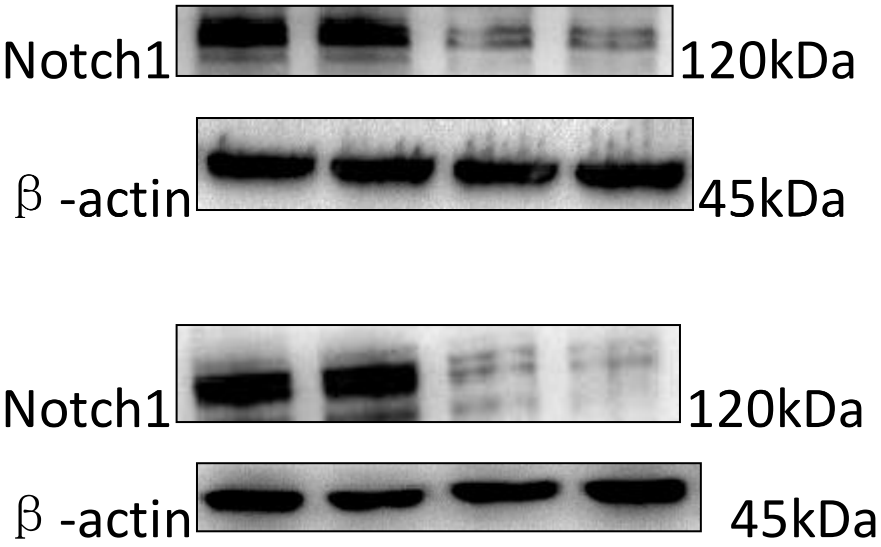

Supplementary Figure S6. Full length blots of data shown in Fig.5A

The gels were initially cut ranged from 45kd to 120 kd.

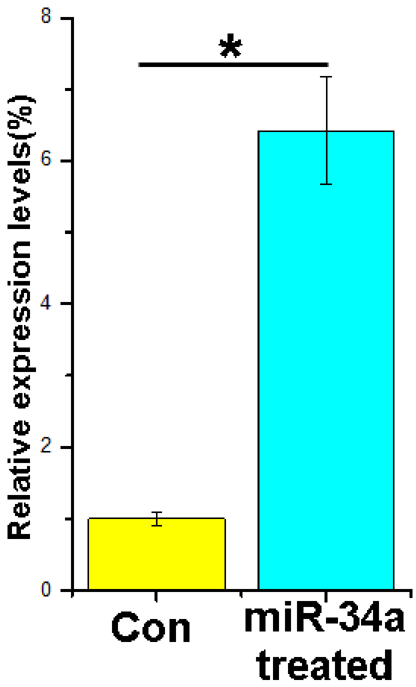

Supplementary Figure S7.The levels of miR-34a in control group and the miR-34a treated group.

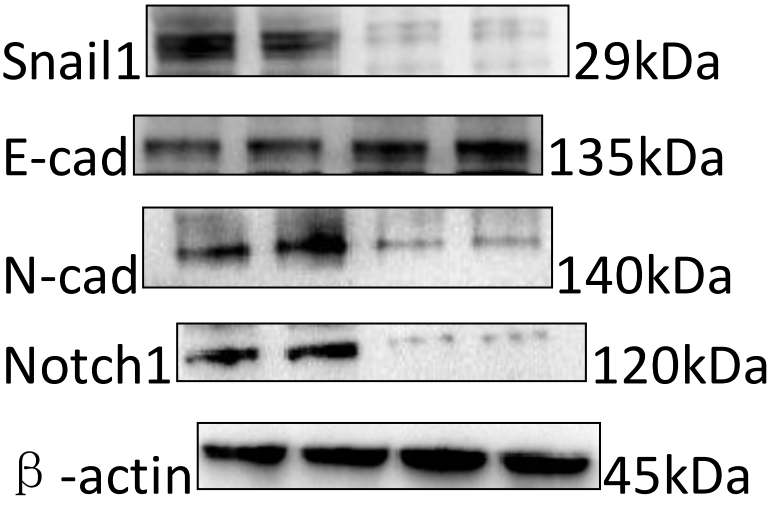

Supplementary Figure S8. Full length blots of data shown in Fig.7C

The gels were initially cut ranged from 29kd to 140 kd.
